# A rapid literature review on the health-related outcomes of long-term person-centered care models in adults with chronic illness

**DOI:** 10.3389/fpubh.2023.1213816

**Published:** 2023-08-21

**Authors:** Fátima Cano, Elisabete Alves, Ana João, Henrique Oliveira, Lara Guedes Pinho, César Fonseca

**Affiliations:** ^1^Local Health Unit of Baixo Alentejo, Beja, Portugal; ^2^São João de Deus School of Nursing, University of Évora, Évora, Portugal; ^3^Comprehensive Health Research Center (CHRC), University of Évora, Évora, Portugal; ^4^Instituto de Telecomunicações, Lisbon, Portugal; ^5^Polytechnic Institute of Beja, Beja, Portugal

**Keywords:** aging, health-related outcomes, long-term care, patient-centered care, self care

## Abstract

**Introduction:**

The strong association between age and the increasing prevalence of chronic diseases, makes it imperative to promote self-care throughout life. Systematic knowledge on the health findings of person-centered care models may contribute to designing effective healthcare strategies to promote empowerment for self-care in long-term care.

**Objective:**

To assess the association between the implementation of person-centered care models that promote self-care training in long-term care and health-related outcomes, among adults with chronic illness.

**Methods:**

A rapid review of the literature was performed following the Cochrane rapid review methodology. The electronic databases CINAHL, MedicLatina, MEDLINE, and Psychology and Behavioral Sciences Collection were searched for randomized experimental studies, published between 2017 and 2022, that implemented interventions based on person-centered models to promote self-care in adults aged ≥18 years with chronic diseases and needing long-term health care. Verification of the eligibility of the articles and the extraction of data were performed by two independent investigators. Quantitative data on the health-related variables assessed were collected and, through narrative synthesis, health outcomes were grouped into individual, institutional and societal levels.

**Results:**

Eight studies, mostly conducted in European countries, were included. All satisfied more than 60% of the methodological quality score. A large variability among studies was found regarding the number of participants, the data collection period and duration of the intervention, the samples selected and the care model implemented. A high number of health-related outcomes (*n* = 17) were analyzed in the studies, using 52 different instruments. The main health-related outcomes were multidimensional, with implications at the individual, institutional and societal levels. The promotion of overall health and wellbeing (*n* = 4), the implementation of patient-centered care models (*n* = 1), the positive and more frequent interactions with health professionals (2), the decrease on staff psychosocial distress (*n* = 1), and the absence of added costs (*n* = 1), while improving family caregivers’ skills (*n* = 1) were the main health-related outcomes described.

**Conclusion:**

There is a need to develop robust experimental studies focused on the views and experiences of all stakeholders and conducted in different countries and cultures. Short-, medium- and long-term health outcomes should be measured using internationally accepted and validated scales for chronic patients.

## Introduction

Self-care is one of the most important dimensions of the health and well-being of populations (1). Thus, collecting data that facilitate a functional diagnosis or a diagnosis of self-care dependence is essential in developing an individual care plan that, in the medium- and long-term, can translate into health gains (2–5).

There is a strong association between age and the increasing prevalence of chronic diseases (6), emphasizing the need to promote of self-care throughout life (7–9). However, the state of greater vulnerability, the occurrence of pathological processes and thus the decreases in a person’s functionality and independence hinders his or her ability to perform self-care, requiring adaptations on the part of the health sectors and at the social level (3, 4, 10). Thus, there is an emerging need to respond to an imbalance between the provision of care to dependent persons and their ability to care for themselves; thus, many older adults and their families turn to long-term care institutions to guarantee necessary care (5).

Long-term care should ensure that people have the opportunity to optimize their abilities throughout the life course while providing a supportive environment and all the care necessary to maintain and promote their functional abilities (11). This care should value individuals as unique beings, taking into account their needs, values and expectations to provide person-centered care (7, 11).

In this context, it is important to design and implement care models that respond to the needs of populations, respecting their particularities to promote quality of life, independence and empowerment of citizens for self-care, considering the physical, cognitive, emotional and social domains (12).

The literature seems to support the benefits that personalized long-term care models exert in the training of self-care, which contribute to greater independence and autonomy in people’s daily lives (13). Other benefits reported include a more positive perception of quality of life and functionality in people with multimorbidity and frailties (9, 14), a reduction of hospitalizations and readmission rates (15), a reduction of polypharmacy (4, 16) and increased patient satisfaction with the perception of quality of care and access to services (17). However, the absence of reviews that directly compare the main health findings of different models of person-centered care hinders the construction of knowledge about the advantages or disadvantages of self-care training. This systematic knowledge may be a relevant tool for designing and developing sustainable and effective integrated and people-centered health care that promotes the empowerment of self-care in long-term care. In addition, the evidence provided might contribute to enriching clinical practices and improving health governance in the context of healthy aging.

The present rapid review aims to assess the association between the implementation of person-centered care models that promote self-care training in long-term care and health-related outcomes, among adults with chronic illness.

## Methods

The present rapid review was developed according to the Cochrane rapid review methodology’ (18) and registered in PROSPERO (International Prospective Register of Systematic Reviews) under the number CRD42023415151.

### Eligibility criteria

As a starting point for the rapid literature review, the research question was formulated based on the PICO mnemonic (19): What are the health outcomes (O) resulting from the implementation of adult-centered care models (I), that promote empowerment for self-care in long-term care (C), in adults diagnosed with chronic disease (P)?

The inclusion criteria outlined for the present study were as follows: (1) target population over age 18 and diagnosed with chronic disease requiring long-term health care; (2) randomized experimental studies published between 2017 and 2022; and (3) implementation of interventions that describe person-centered models to promote self-care.

The exclusion criteria were as follows: (1) observational studies; (2) nonoriginal studies (reviews, meta-analyses, study protocols, commentary, editorials, journal articles, conference proceedings and abstracts, reports, guidelines and gray literature and scale validations); (3) studies that did not report data on the implementation of interventions that described person-centered models; (4) studies focusing on children or adults without chronic disease and/or without need for long-term health care; and (5) articles written in languages other than English, French, Spanish and Portuguese.

### Data sources

In December 2022, all articles published between 2017 and 2022 in EBSCO’s electronic databases were analyzed using the sources *Academic Search Complete; CINAHL Plus; MedicLatina; MEDLINE* and *Psychology and Behavioral Sciences Collection*.

### Literature search

The following search expression was used in all databases: (long term OR nursing home OR residential care) AND (person centered care OR patient centered care OR client centered care) AND (care models OR models of care OR models). Additionally, the bibliographic references of the articles considered eligible for inclusion were also screened and, whenever the title appeared to be related to the search term, analyzed (*n* = 2). No additional papers were added after full-text analysis. The period from 2017 to 2022 used for literature search, was selected in response to the need to access up-to-date information on the topic studied, since previous literature advocate that systematic literature reviews should take into account evidence from the last 5 years (20, 21).

### Study selection

The identification and selection of studies was carried out according to the proposed objective. All articles were analyzed by two evaluators (FC and AJ), who independently examined all the articles obtained, first based on the title and abstract and second based on the full texts. Publications with titles and abstracts without adequate information to determine whether they met inclusion/exclusion criteria were subjected to full-text review. Discrepancies were discussed among the authors until a consensus was reached. When consensus could not be reached, a third rater (EA) resolved the conflict.

### Data extraction

A data extraction sheet was specifically developed for this study and completed by two independent raters (FC and EA). Descriptive data were collected to characterize the studies, including information on the authors and year of publication; country where the study was conducted; period of data collection; setting; participants and sample; study design and data collection methods. Specifically regarding the interventions implemented data on the intervention name, content, main providers, delivery mode and frequency, and the instruments used to evaluate the health-related outcomes of the interventions were also withdrawn. The health outcomes of the person-centered care models to promote empowerment for self-care described in each study were identified and the main results were retrieved. Quantitative data on health-related variables whose association with the interventions was statistically significant were also collected, and the directions of the associations were recorded. All other variables whose association with the intervention was tested and reported were also extracted.

### Methodological quality assessment

The articles were subjected to evaluation of methodological quality and levels of evidence of JBI Critical Appraisal Tools (22). The JBI checklist score was as follows: “Yes” with 1 point, “No” and “Unclear” with 0 points. Scores were given for adherence to each of those aspects, ranging from 0 (poorly conducted randomized controlled trial) to 13 (excellent conducted randomized controlled trial). Decisions regarding methodological quality of the studies included were made, independently, by two reviewers, and any disagreements were resolved by discussion.

The sum of the points was classified as the percentage of the items present, considering the recommendations of the authors Camp and Legge (23). Thus, a score between 70 and 79% of the checklist criteria was classified as medium high quality, between 80 and 90% was assigned high quality, and a score greater than 90% of the criteria was classified as excellent quality. Evidence levels were classified according to the JBI (24).

### Data synthesis

Mean differences with the respective 95% Confidence Intervals (CI) or *p*-values on the effectiveness of interventions were collected, whenever available, and the directions of the associations were registered. Subsequently, through narrative synthesis (25), it was possible to synthesize and aggregate the content in the different categories based on the similarity of meaning, and health outcomes were grouped into three levels: individual (including characteristics related to promoting the participants’ physical and psychological well-being), institutional (including available resources, care provision and variables related to health professionals) and societal (including characteristics related to the impact of interventions on society).

## Results

### Description of study selection and study characteristics

**Figure 1:**
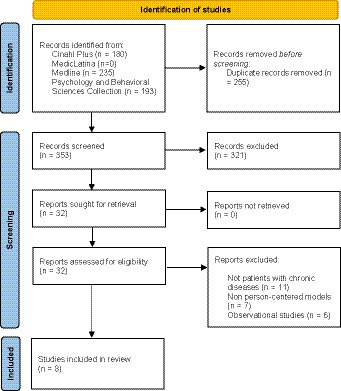
Systematic literature review flowchart.

The search results and the screening process of this rapid review are presented in detail in the flowchart in Figure 1 (26). The search expression resulted in a total of 608 articles, and after eliminating duplicate results (*n* = 255), 352 articles were examined. Of these, 321 articles were eliminated because they were observational studies (*n* = 286), non-original full-length studies (*n* = 23), do not included patients with chronic diseases (*n* = 7), do not implemented person-centered models (*n* = 3), or the study population were aged bellow 18 years old (*n* = 2). Of the 32 articles selected for full-text analysis, 11 were excluded because they were not conducted with people diagnosed with chronic disease observational studies, 7 because they did not use person-centered care models, and 6 because they were not conducted with people diagnosed with chronic disease, thus resulting in a total of 8 articles being included in this rapid review (27–34).

The main characteristics of the 8 studies included are described in Table 1. The studies were conducted mostly in European countries (*n* = 6), namely, the United Kingdom (*n* = 3). Only one study was conducted in the Americas (32), and another was conducted in Asia (34). The data collection periods occurred between 2013 and 2021, varying between 8 (28) and 46 months (33). The follow-up period of the participants ranged between 9 (27) and 21 months (31), and half of the studies (*n* = 4) were conducted in nursing homes. Cluster-randomized controlled trial was the most frequent study design (*n* = 5). Only one study implemented a step wedge research design, comparing two models of dementia-specific case conferences (31).

**Table 1:** Overall description of the studies included (*n* = 8).

Publication	Country	Period of data collection	Setting	Participants and sample	Study design	Data collection methods
(27)	United Kingdom	January 2013–September 2015 (9 months follow-up)	69 Nursing homes	People with dementia (n = 847)	Cluster-randomized controlled trial	Survey (outcome measures were assessed prior to randomisation and after 9 months of the intervention)
(28)	United Kingdom	May 2015–December 2015 (15 months follow-up)	33 General practices providing National Health Service primary medical care	Patients with multimorbidity (*n* = 1,546)	Cluster-randomized controlled trial	Survey (outcomes were collected at baseline and 9 and 15 months after recruitment)
(29)	United Kingdom	January 2015–February 2016 (12 months follow-up)	Four centers in England and Wales; one center in Scotland	Adults aged ≥18 years with heart failure with reduced ejection fraction (*n* = 216)	Multicenter randomized trial	Survey (outcomes were collected at baseline, and 4, 6 and 12 months after recruitment)
(30)	The Netherlands	May 2016–December 2018 (12 months follow-up)	19 Nursing home residences	Residents with dementia (*n* = 231)	Cluster-randomized controlled trial	Survey (outcomes were collected at baseline, and 1, 3, 6 and 12 months after baseline)
(31)	Germany	September 2013–April 2015 (21 months follow-up)	12 Nursing homes	People with dementia (*n* = 224)	Stepped-wedge cluster randomized design	Survey [outcomes were collected every 3 months at seven data points (T0–T6)]
(32)	United States	2020–2021 (12 months follow-up)	55 Nursing homes	Adults aged ≥55 years with cognitive impairment (*n* = 553)	Cluster-randomized controlled trial	Survey (outcomes were at baseline, and 6 and 12 months post implementation)
(33)	Denmark	February 2016–November 2019 (12 months follow-up)	Rheumatology rehabilitation center	Adults aged ≥18 years with chronic low back pain (*n* = 165)	Randomized controlled trial	Survey [outcomes were collected at baseline (before randomization), before intervention start, and 6 and 12 months after the start of the rehabilitation program]
(34)	Taiwan	2018–2019 (12 months follow-up)	3,000-Bed medical center	Adults aged ≥60 years with hip fracture and cognitive impairment (*n* = 152)	Randomized controlled trial	Survey (outcomes were assessed within 4 h after hospital admission, after surgery, before discharge and, 1, 3, 6, and 12-months after discharge)

### Participants characteristics

The sample size ranged from 152 (34) to 1546 (28) participants diagnosed with dementia (27, 30, 31), multimorbidity (28), heart failure (29), cognitive impairment (32), chronic low back pain (33) or hip fracture (34) (Table 1).

### Interventions characteristics

All studies implemented a structured rehabilitation program based on person-centered models of care to promote empowerment for self-care in the context of long-term care, and the majority compared each intervention results with the provision of usual care (*n* = 7), as described in Table 2. Interventions were mainly provided by health professionals, namely nurses (27–30, 33, 34). All the interventions were delivered face-to-face, with only 1 also adding telephone contacts during the implementation (29), with a highly variable frequency, ranging from 7-days-a-week (30) to every 2 months’ meetings (34).

**Table 2:** Main characteristics of the interventions implemented to promote empowerment for self-care.

Publication	Intervention name	Intervention content	Intervention providers	Intervention delivery mode and frequency	Health-related outcomes (instruments)	Main results of the intervention
(27)	WHELD intervention	Staff training in person-centered care, social interaction, and guidance on use of antipsychotic medications versus treatment as usual	1 Full-time research therapist, and 2 lead care staff members in each care home	Orientation phase: Face-to-face (2 whole days or 4 half days in each home over 1 month) Intervention delivery phase: Face-to-face (6–8 h per month, during 8 months)	Primary outcome: Quality of life (DEMQOL-Proxy^1^) Secondary outcomes: Agitation (CMAI^2^) Neuropsychiatric symptoms (NPI-NH^3^) Dementia severity (CDR^4^) Global deterioration (CDR^4^) Functional Assessment (FAST^5^) Mood (CSSD^6^) Unmet needs (CANE^7^) Quality of interactions (QUIS^8^) Pain (Abbey Pain Scale) Antipsychotic use Mortality Costs (intervention, accommodation, and health and social care costs)	Primary outcome: Improvement in QoL [mean difference (95%CI): 2.54 (0.81 to 4.28)] Secondary outcomes: Reduction on agitation (*p* = 0.008) and on overall neuropsychiatric symptoms (*p* < 0.001) Increase in the proportion of positive care interactions (*p* = 0.030) Reduction of health and social care cost [mean difference (95%CI): −4.74.54 (−6.13 to −3.16)]
(28)	3D approach	3D reviews (nurse, pharmacist and physician) on health, depression, and drugs dimensions versus usual care (review of chronic conditions by a nurse)	Nurses and physicians, from the practice staff, and pharmacists	Clinical staff training: Face-to-face (2 half-days) Intervention delivery: Face to face (6-monthly comprehensive 3D reviews)	Primary outcome: QoL (EQ-5D-5 L^9^) Secondary outcomes: Ilness burden (Bayliss measure and HADS^10^). Treatment burden (MTBQ^11^; MMAS^12^, and number of drugs prescribed). Patient-centred care (PACIC^13^, CARE^14^, NHS LTCQ^15^, and NHS GP Patient Survey^16^). Continuity of care (COCI^17^, Visit Entropy measure, and numbers of consultations in primary and secondary care). Disease management (United Kingdom QOF^18^, and high-risk prescribing assessment)	Primary outcome: No evidence of a difference in health-related QoL [mean difference (95%CI): 0·00 (−0·02 to 0·02)] Secondary outcomes: Improvement on patient-centred care measures: PACIC (*p* < 0.001), CARE doctor (*p* < 0.011), and CARE nurse (*p* = 0.044). Increase on the proportion of patients reporting care related to their priorities (*p* < 0.001), those reporting care as joined-up (*p* < 0.001), those reporting a written care plan (*p* = 0.001), and overall satisfaction with care (*p* = 0.001). Higher number of nurse (*p* < 0.001) and primary care physician consultations (*p* = 0.021)
(29)	REACH-HF intervention	Facilitated self-care and home-based cardiac rehabilitation plus usual care versus usual care (no cardiac rehabilitation approach that included medical management)	Trained health-care professionals with specialist cardiac experience	Staff training: Face-to-face (three-day training course) Intervention delivery: Face-to-face (*n* = 3) and telephone contacts (*n* = 4) over 12 weeks	Primary outcome: Disease-specific QoL (MLHFQ^19^) Secondary outcomes: Generic QoL (EQ-5D-5 L^9^, HeartQoL questionnaire). Psychological wellbeing (HADS^10^) Exercise capacity (ISWT^20^) Physical activity (GeneActiv accelerometer) Self-care (SCHFI^21^) Mortality Hospitalization	Primary outcome: Improvement in the overall score of disease-specific QoL [mean difference (95%CI): 5.7 points (0.7–10.6)] Secondary outcomes: Improvement on self-care (*p* < 0.001)
(30)	Namaste care family program	Multidimensional care program with psychosocial, sensory and spiritual components versus usual care	Nursing staff and volunteers	Intervention delivery: Face-to-face (7-days-a-week in two 2-h sessions over 12 months)	Primary outcome: QoL (QUALID^25^ and EQ-5D-3 L^26^) Family caregiver gains (GAIN^27^) Secondary outcome: - Quality Adjusted Life Year (amount of time a participant spent in a specific health state multiplied by the utility score associated with that health state) Costs (secondary care, medication, family and intervention program costs)	Primary outcome: Differences in QUALID and GAIN scores between the Namaste Care Family program and usual care were small and statistically not significant (Mean difference (95%CI): −0.060 (− 0.42 to 0.30) and 0.033 (−0.24 to 0.31), respectively)
(31)	FallDem	Two models of dementia-specific case conferences: sequential transition from control (usual care) to intervention conditions	Project team	Training: In-service training (6 h) plus supervised training on the job (3 h) Sessions: Four face-to-face case conferences (1 h to 1.5 h each, during 7-month)	Primary outcome: Behaviour that challenges (NPI-NH3) Secondary outcomes: QoL (QUALIDEM) Psychotropic medications Caregiver burnout (CBI^22^) Work-related stress (BelaDem^23^) Vocational action competence (KRI^24^)	Primary outcome: No significant changes on behaviour that challenges [Mean difference (95%CI): 0.03 (−0. 09 to 0.14)] Secondary outcomes: Positive and negative statistically significant changes for particular subscales of quality of life were found, but the differences were very small and not clinically relevant Reduction in the mean score of work-related burnout (*p* = 0.032)
(32)	EIT-4-BPSD	Evidence Integration Triangle for Behavioral and Psychological Symptoms of Distress in dementia vs. usual care (only education of the staff)	Research facilitators	Environment and policy assessments: Face-to-face session (*n* = 1) Education of the staff: Face-to-face session (*n* = 1) Person-centered care plans for BPSD: Face-to-face sessions (*n* = 4 to 11) Mentoring and motivating staff: Face to face monthly meetings for 12-months (*n* = 12)	Functionality (Barthel Index) Depression (CSDD^28^) Agitation (CMAI^2^) Resistiveness to care (RTC^29^) Psychotropic medications Pain (PAINAD^30^) QoL(QUALID^25^) Care community outcomes (Environment Assessment for Person-Centered Management of BPSD; Policy Assessment for Person-Centered Management of BPSD, and Checklist for Evidence of Person-Centered Approaches for BPSD in Care Plans) Staff-Patient interactions (QUIS^8^)	No significant treatment effect for any of the outcomes assessed.
(33)	Integrated multidisciplinary rehabilitation programme	Integrated programme (a pre-admission day, 2 weeks at home, 2 weeks’ inpatient followed by home-based activities, plus two 2-day inpatient booster sessions, and six-month follow-up visit) versus Existing programme (four-week inpatient, and six-month follow-up visit)	Multidisciplinary team (physiotherapists, occupational therapists, nurses, rheumatologist, nutritional counsellor)	Face to face (14 weeks, with approximately 50 contact hours)	Primary outcome: Back-specific disability (ODI^31^) Secondary outcomes: Back pain intensity (NRS^32^) Pain self-efficacy (PSEQ^33^) Health-related QoL (EQ-5D 5L^9^) Depression (MDI^34^)	Primary outcome: No statistically nor clinically significant change in back-specific disability [mean difference (95%CI): −0.53 (−4.08 to 3.02)]
(34)	Family-centered care model	Family-centered care intervention after hip fracture (geriatric assessment, discharge planning, in-home rehabilitation, and family caregiver-training for dementia care) versus usual care (no in-home programs for rehabilitation or nursing care)	Geriatric nurses	Geriatric assessment: Face to face Discharge planning: Face-to-face Rehabilitation program: During hospitalization (daily); In-home rehabilitation of 30 min (weekly during the 1st month, every 2 weeks in the 2nd and 3rd months, every month from the 4th to 6th months, and every 2 months from the 7th to 12th months, after discharge) Family caregiver-training: 2 face-to-face sessions (1 and 1.5 months after discharge)	Activities of daily living (Barthel Index and iADL^35^) Nutritional status (MNA Elderly^36^) Health-related quality of life (SF-36^37^) Self-rated health (EQ-5D^9^) Cognitive status (MMSE^38^) Caregiver self-efficacy (AMSS^39^) Caregiver competence (CCS^40^)	Improvement in self-rated health (*p* < 0.05) and nutritional status (*p* < 0 0.05) Family caregivers had a higher level of competence (*p* < 0.01), and greater rates of improvement in competence (*p* < 0.01) and self-efficacy (*p* < 0.05)

QoL, quality of life; BPSD, behavioral and psychological symptoms of distress. ^1^Dementia quality of life measure; ^2^Cohen-Mansfield Agitation Inventory; ^3^Neuropsychiatric Inventory-Nursing Home Version; ^4^Clinical Dementia Rating; ^5^Functional Assessment Staging Tool; ^6^Cornell Scale for Depression in Dementia; ^7^Camberwell Assessment of Need for the Elderly; ^8^Quality of Interaction Survey; ^9^Five-level version of the EuroQol-5 Domain; ^10^Hospital Anxiety and Depression score; ^11^Multimorbidity Treatment Burden Questionnaire; ^12^Morisky Medication Adherence eight-item score; ^13^Patient Assessment of Care for Chronic Conditions; ^14^ Consultation and Relational Empathy; ^15^NHS Long Term Conditions 6 questionnaire; ^16^NHS General Practice Patient Survey; ^17^Continuity of Care index; ^18^UK Quality and Outcomes Framework; ^19^Minnesota Living with Heart Failure Questionnaire; ^20^Incremental shuttle walk test; ^21^Self-Care of Heart Failure Index; ^22^Copenhagen Burnout Inventory; ^23^Dementia-Specific Burden instrument; ^24^Competence-Reflection Inventory; ^25^Quality of Life in Late-Stage Dementia; ^26^Three-level version of the EuroQol-5 Domain; ^27^Gain in Alzheimer Care Instrument; ^28^The Cornell Scale for Depression in Dementia; ^29^Resistance to Care Scale; ^30^Pain in Advanced Dementia Scale; ^31^Oswestry Disability Index; ^32^Numerical Rating Scale; ^33^Pain Self-Efficacy Questionnaire; ^34^Major Depression Inventory; ^35^Lawton-Brody Instrumental Activities of Daily Living; ^36^Mini Nutritional Assessment; ^37^36-Item Short Form Survey; ^38^Mini Mental State Examination; ^39^Agitation Management Self-Efficacy Scale; ^40^Caregiver Competence Scale.

### Methodological quality of studies

The evaluation of the methodological quality and levels of evidence of the studies, according to the JBI Critical Appraisal Tool (22), concluded that all the studies satisfied more than 60% of the proposed quality criteria (Supplementary Table S1). The average quality score of the studies was 10 out of 13 (ranging from 8 to 11; Table 3). Most of the studies have medium high methodological quality (28, 31, 32, 34), and two have high quality (27, 29). Data regarding the concealment of the allocation of the treatment groups were unclear in most studies and, given their nature, none was able to blind those delivering treatment to treatment assignment. However, more than half (*n* = 5) reported blinding the participants and the outcomes assessors to treatment assignment. All included studies are evidence level 1.c - randomized controlled trial (Table 3).

**Table 3:** Quality score of JBI critical appraisal checklist.

Publication	JBI Quality score (22)	Level of evidence (24)
(27)	11 (85%)	1.c
(28)	10 (77%)	1.c
(29)	11 (85%)	1.c
(30)	8 (62%)	1.c
(31)	10 (77%)	1.c
(32)	10 (77%)	1.c
(33)	9 (69%)	1.c
(34)	10 (77%)	1.c

*Higher values indicate well conducted randomized controlled trial (range: 0–13).

### Health related outcomes characteristics

The health-related outcomes assessed were highly variable including characteristics related to the participants’ physical and psychological health and wellbeing, the provision of care, health professionals’ competence and wellbeing, caregivers’ self-efficacy and economic and social costs (Table 2). Quality of life, the most frequent primary outcome assessed (*n* = 4), was the only health-related variable considered in all studies, being measured by 7 different instruments. Overall, 17 different health-related variables were analyzed in the studies, resorting to 52 different instruments. In fact, the instruments used to assess the health outcomes of the interventions were highly variable, with the EuroQol-5 Domain being the only scale used repeatedly in five studies (28–30, 33, 34). The Hospital Anxiety and Depression score (HADS), the Quality of Life in Late-Stage Dementia (QUALID), the Neuropsychiatric Inventory-Nursing Home Version (NPI- NH), the Quality of Interaction Survey (QUIS), the Barthel Index and the Cohen-Mansfield Agitation Inventory (CMAI) were used in two different studies each.

All the studies used structured questionnaires to gather quantitative data from the participants. Few studies described statistically significant and clinically relevant results for most of the health-related outcomes of person-centered care models to promote empowerment for self-care, with only three studies finding significant positive results regarding their primary outcome (27, 29, 34) (Table 2).

### Effects of interventions on the health-related outcomes

Table 4 presents a synthesis of the health-related results of the person-centered care models to promote self-care empowerment in the included studies. The results were grouped into three levels of analysis: individual, institutional and societal. Factors related to the characteristics of individuals were addressed most frequently, followed by institutional and societal factors.

**Table 4:** Synthesis of the associations described for the health-related outcomes of person-centered care models to promote empowerment for self-care, at the individual, institutional and societal levels.

	Direct association	Inverse association	No association
Individual level
Overall health^1^	✓ (34)	–	✓ (27)
Pain	–	–	✓✓✓ (27, 32, 33)
Functionality	–	–	✓✓✓✓ (27, 32–34)
Physical capacity^4^	–	–	✓✓ (29, 34)
Nutritional status	✓ (34)	–	–
Psychological wellbeing^2^	✓ (29)	-	✓✓✓✓✓ (28, 29, 32–34)
Neuropsychiatric symptoms^3^	–	✓ (27)	✓✓ (31, 32)
Quality of life	✓✓✓ (27, 29, 31)^a,b^	✓ (31)	✓✓✓✓✓✓ (28–30, 32–34)^d^
Institutional level
Health care resources^5^	✓✓ (27, 28)	-	-
Staff competence^6^	-	-	✓ (31)
Staff psychosocial wellbeing^7^	-	✓ (31)^e^	✓ (31)^f^
Treatment burden^8^	-	-	✓✓✓✓ (27, 28, 31, 32)
Patient-centered care	✓ (28)	-	✓ (32)
Hospital admissions	–	-	✓✓✓ (28, 29, 34)
Societal level
Costs^9^	-	✓ (27)	✓✓✓ (29, 30, 34)
Mortality	-	-	✓✓✓✓ (27–29, 34)
Family caregiver gains^10^	✓ (34)	-	✓ (30)

^1^Self-rated health and global deterioration; ^2^Anxiety, depression, self-care and the effect of illness on life; ^3^Delusions, hallucinations, agitation, dysphoria, apathy, irritability, euphoria, disinhibition, aberrant motor behavior, night-time behavior disturbances, mood, behavioral changes, and eating abnormalities; ^4^Exercise capacity, physical capacity and physical-related health outcomes; ^5^Nurse consultations, primary care physician consultations, staff and patient interactions; ^6^Vocational and methodological expertise, social competence and self-competence; ^7^Burnout and work-related stress; ^8^Number of prescribed drugs, medication adherence, psychotropic medication and overall disease management; ^9^Health care and social costs; ^10^Overall gains, competence and self-efficacy. ^a^Health-related QoL; ^b^Care relationship, positive affect and social isolation dimensions for severe dementia, and having something to do dimension for less severe dementia; ^c^Positive affect, positive relations and self-image dimensions for less severe dementia; ^d^Generic QoL; ^e^Work-related burnout; ^f^Work-related stress.

At the individual level, studies reported a direct association between global health, particularly regarding self-rated health (34), and the intervention implemented. However, there was no significant association with general dementia deterioration (27), pain relief (27, 32, 33), functionality (27, 32–34) or physical capacity (29, 34). Nutritional status was positively and significantly associated with the intervention performed (34). Regarding psychological well-being, a positive result was found between self-care and the intervention performed (29), suggesting that the implementation of person-centered care models to promote empowerment for self-care meets its objectives. However, the evaluation of depression, anxiety and the effect of the disease on the lives of the participants did not seem to change after the intervention (28, 29, 32–34). While one study concluded that the intervention, which combined the training of health professionals, social interaction and the use of antipsychotic medication, was effective in reducing neuropsychiatric symptoms (27), other studies reported the absence of an association with behavioral changes or agitation (31, 32). Quality of life was the only health outcome analyzed in all studies, presenting contradictory results. Although some studies seem to conclude that after the implementation of care models, there were benefits for the participants’ quality of life (27, 29, 31), others described an inverse association in patients with less severe dementia regarding positive affect, positive relationships and self-image dimensions (31). However, more than half of the studies lacked statistical evidence to support the benefits of interventions on the participants’ perception of quality of life (Table 4).

Health resources, namely, medical and nursing consultations, and the interaction between health professionals and patients were the main health outcomes directly associated with interventions (27, 28) at the institutional level (Table 4). At the same time, the professionals’ perception of competence, the number of hospital admissions, and the *burden* of treatment did not show any significant association after the implementation of the intervention. One of the studies that evaluated the employment of patient-centered care reported a direct association with empowerment for self-care (28), while another highlighted the absence of significant associations. Data on the psychosocial well-being of the staff showed inconsistent results in the same study, according to the assessed dimension (31). While for work-related *burnout* the association described with the intervention was inverse, for work-related stress, there was no statistical evidence of any association.

Data on interventions that assessed health outcomes at the societal level revealed mostly non-significant results. Only one study reported a significant decrease in total health and social care costs compared to usual care (27), while the remaining study concluded that there were no differences in the costs associated with providing care. Similarly, no significant associations were found regarding the number of deaths (27–29, 34). Regarding gains for family caregivers, studies highlight a statistically positive association with the self-efficacy and competence of caregivers (34) (Table 4).

## Discussion

The present rapid review of the literature revealed that the main health-related outcomes resulting from the implementation of person-centered care models to promote empowerment for self-care in long-term care are multidimensional, with implications at the individual, institutional and societal levels. The results highlight the variety of interventions implemented, as well as the exploitation of a wide range of health outcomes, evaluated through different instruments.

At the individual level, the promotion of quality, psychological well-being, nutritional status and health perception of the participants, as well as the reduction of neuropsychiatric symptoms, are the main benefits of the implemented models. Although the literature supports the associations described (3, 9, 13, 35), the systematic analysis of the studies revealed inconsistent results among them. Although some articles supported a positive association between the interventions and the participants’ perception of quality of life, psychological well-being and general perception of health, others did not find scientific evidence that clearly supports these conclusions. The heterogeneous evaluation of these characteristics, namely regarding the variability of the instruments used, compromises the comparability of the results between the different studies and may partially justify the contradictory results. Additionally, the lack of specific and validated instruments to evaluate people with chronic diseases in need of long-term care may lead the studies to neglect specific dimensions of mental health, quality of life and well-being that are particularly relevant for people with chronic disease that may not be addressed by instruments aimed at the general population. The inverse association with neuropsychiatric symptoms reported in the study by Ballard et al. (27) was not confirmed by two other studies. However, these two studies focused essentially on two neuropsychiatric symptoms, unlike the previous study that reported data on a combination of 12 neuropsychiatric disorders. Characterizing neuropsychiatric symptoms is a challenge, and there is a pressing need to resort to instruments that assess a broader range of behavioral symptoms (36). This will help health professionals and researchers to detect, quantify, and monitor neuropsychiatric symptoms in a more integrated manner that is appropriate to the needs of each person (37).

Contrary to what has been described in the literature, improvements in the functionality and the physical capacity of participants did not reach statistical significance after the implementation of the interventions. Functioning is assumed to be a central variable in the aging process, a dynamic interaction between health states and contextual factors (environmental and personal) (38) that results in the dependence/independence of each individual (39). The absence of significant results in these dimensions may be due to the context of long-term care in which the interventions were implemented. Often, the request for long-term care is made by families when a patient has reached an advanced stage of functional dependence or when there is an imbalance between the care needs and the necessary and appropriate responsiveness in the family context (4, 40, 41). Thus, future studies should evaluate different levels of care, allowing for a stratified analysis of participants’ functionality by level of care.

All studies implemented a structured, supervised, individualized and personalized intervention program according to the needs of each person and his or her circumstances. Previous literature supports that the involvement of patients with chronic disease in interventions that promote self-care facilitates the concrete perception of their preferences and abilities (42, 43), motivating them to promote and participate in their self-care (3, 10, 13, 44). Although only one study (28) reported benefits of implementing patient-centered care for the promotion of self-care, the other study that also analyzed this variable concluded that there was a slight, but not significant, benefit to the implementation of policies and environments that supported person-centered approaches to dementia care (32). The difficulty in finding significant results regarding the implementation of person-centered care models may be due to a long-standing problem faced by care services regarding the lack of an adequate and properly paid workforce, which would ensure the implementation of quality person-centered care (4, 45–47).

The interventions developed favored a significant increase in the proportion of positive interactions between the team of health professionals and patients with chronic disease (27), as well as in the number of consultations with nurses and primary care physicians (28). These findings emphasized the previously reported importance of the benefit conferred by social interaction and pleasurable activities in empowerment for self-care (48). This greater social interaction may also be beneficial for health professionals, as the results reveal a decrease in risk factors for work-related *burnout*. Thus, it is important to optimize care and interventions to improve care, promote interdisciplinarity and reduce the burden of the health team to promote the health and well-being of both patients and health professionals (49, 50).

Although only one study reported a significant decrease in total health and social care costs compared to usual care, the remaining studies that evaluated this dimension concluded that the implementation of interventions did not exacerbate the costs associated with providing care. This is a relevant indicator to consider in the design of health policies since these results seem to suggest that strategies to promote empowerment for self-care (51–53) show health gains without added costs to the health and social systems. Similarly, the absence of significant differences in mortality is interpreted as a positive finding, not compromising the implementation of the intervention. Finally, the implementation of care models to promote self-care seems to have a positive impact on caregivers, contributing to improving their self-efficacy and competence without increasing their perceived *burden*. In fact, family-centered intervention programs are beneficial for increasing the confidence, knowledge and skills of family caregivers of chronically ill patients (54, 55). The inclusion of informal caregivers in care provision is particularly important, as they are often challenged by a lack of information sharing, frequent role confusion and disorganized care planning (56, 57).

To the best of our knowledge, this is the first review that aims to analyze the health-related outcomes of randomized trials that evaluate the effect of different models of care centered on people with chronic disease, performed in long-term care, with the objective of promoting empowerment for self-care. However, some limitations must be discussed. The period from 2017 to 2022 used for literature search, was selected in response to the need to access the most up-to-date information on the topic being researched. Although some relevant papers published before 2017 may have been excluded from the current analysis, previous literature suggests that the conclusions of most systematic reviews might be valid for approximately 5 years (21). Thus, it has been advocated that systematic literature reviews should take into account evidence from the last 5 years (20), which should reflect the state of current knowledge on the field. Additionally, there was considerable heterogeneity among the studies, particularly regarding the specific characteristics of the selected samples, the care model implemented, the variety of scales used and the diversity of health outcomes evaluated. However, the selected databases, the search strategy and the inclusion criteria were carefully structured and supported by the literature and research experiences to capture the largest number and diversity of studies to meet the objectives of this rapid review of the literature.

## Conclusion

**Figure 2:**
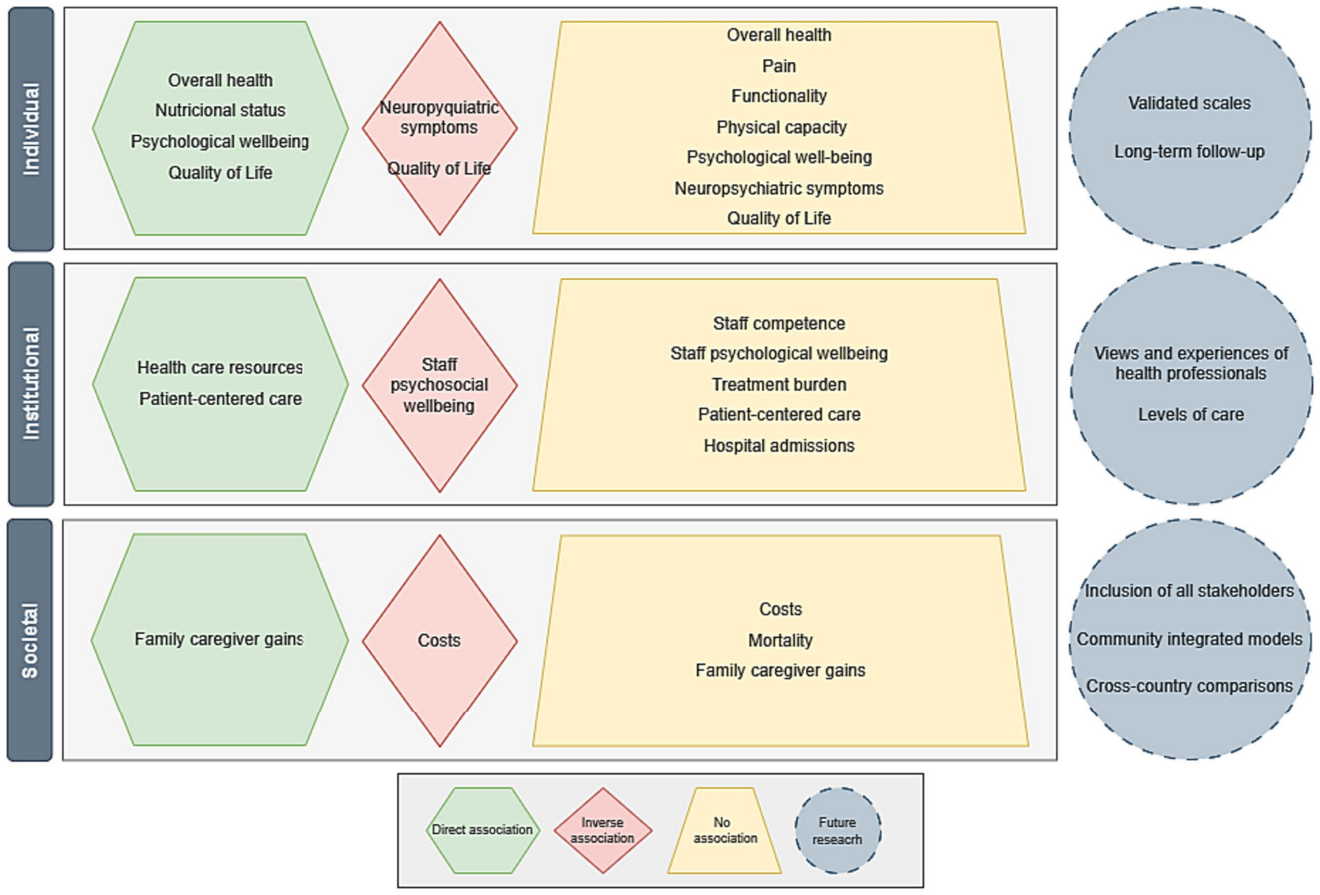
Map of the health-related outcomes of person-centered care models to promote empowerment for self-care.

A comprehensive mapping the health-related outcomes of person-centered care models to promote empowerment for self-care is presented in Figure 2, to synthetize our main conclusions. The absence of statistically significant associations for a large proportion of health outcomes emphasizes the need to develop robust experimental studies conducted in different countries and cultures, using internationally accepted and validated scales for chronic patients to assess health gains at short-, medium- and long-term. Such may contribute to identifying at-risk groups, empower the chronically ill to assume self-care, and promote the main facilitators for self-care. Also, the inclusion of the views and experiences of health professionals and the analysis of health outcomes by level of care may enhance the promotion of health and well-being among health professionals, while adapting the interventions to meet the needs of patients. Finally, the design and implementation of integrated care models in the community that promote the integration of variables that evaluate the interpersonal relationships between chronically ill patients, their peers, family members and caregivers, as well as the cooperation of health and social services will contribute to the integration of policies focused on the needs and experiences of citizens. Also, comparisons between countries will enhance the capture of broader experiences, diverse and representative, allowing comparisons and providing quality support to all stakeholders, taking into account different realities, needs and cultural origins.

## Author contributions

FC and AJ reviewed the literature and identified the studies. FC and EA selected the studies and were responsible for the data extraction. FC drafted the manuscript. AJ, HO, and LP collaborated in the analysis and interpretation of the data and critically reviewed the manuscript. CF and EA designed the study, analyzed and interpreted the data, and critically reviewed the article for important intellectual content. All authors contributed to the article and approved the submitted version.

## Funding

This research was funded by the European Regional Development Fund (ERDF) through the Program Interreg VA España-Portugal (POCTEP) 2014–2020, International Institute for Research and Innovation in Aging–Capitaliza, “0786_CAP4ie_4_P”. This work was funded by national funds through the Foundation for Science and Technology, under the project UIDP/04923/2020.

## Conflict of interest

The authors declare that the research was conducted in the absence of any commercial or financial relationships that could be construed as a potential conflict of interest.

## Publisher’s note

All claims expressed in this article are solely those of the authors and do not necessarily represent those of their affiliated organizations, or those of the publisher, the editors and the reviewers. Any product that may be evaluated in this article, or claim that may be made by its manufacturer, is not guaranteed or endorsed by the publisher.
