# Supplementary material for: A rapid literature review on the health-related outcomes of long-term person-centered care models in adults with chronic illness
Source: Front Public Health. 2023 Aug 21;11:1213816. doi: 10.3389/fpubh.2023.1213816 (PMC10477001; doi:10.3389/fpubh.2023.1213816)
Supplement: Supplementary file 1 [file Table_1.docx]

Supplementary Table. Critical Appraisal for the included studies.

| **JBI critical appraisal checklist for randomized controlled trials** | **Ballard, et**  **al., 2018** | **Salisbury, et al., 2018** | **Dalal, et. al,**  **2019** | **El Alili et al., 2020** | **Halek, et al.,**  **2019** | **Resnick, et al., 2021** | **Schmidt, et al.,**  **2021** | **Tseng, et al.,**  **2021** |
| --- | --- | --- | --- | --- | --- | --- | --- | --- |
| 1. Was true randomization used for assignment of participants to treatment groups? | 1 | 1 | 1 | 0 | 1 | 1 | 1 | 1 |
| 2. Was allocation to treatment groups concealed? | 0 | 0 | 0 | 0 | 0 | 0 | 0 | 0 |
| 3. Were treatment groups similar at the baseline? | 1 | 1 | 1 | 1 | 1 | 1 | 1 | 1 |
| 4. Were participants blind to treatment assignment? | 1 | 0 | 1 | 0 | 1 | 1 | 0 | 1 |
| 5. Were those delivering treatment blind to treatment assignment? | 0 | 0 | 0 | 0 | 0 | 0 | 0 | 0 |
| 6. Were outcomes assessors blind to treatment assignment? | 1 | 1 | 1 | 0 | 1 | 1 | 0 | 0 |
| 7. Were treatment groups treated identically other than the intervention of interest? | 1 | 1 | 1 | 1 | 1 | 0 | 1 | 1 |
| 8. Was follow up complete and if not, were differences between groups in terms of their follow up adequately described and analyzed? | 1 | 1 | 1 | 1 | 1 | 1 | 1 | 1 |
| 9. Were participants analyzed in the groups to which they were randomized? | 1 | 1 | 1 | 1 | 1 | 1 | 1 | 1 |
| 10. Were outcomes measured in the same way for treatment groups? | 1 | 1 | 1 | 1 | 1 | 1 | 1 | 1 |
| 11. Were outcomes measured in a reliable way? | 1 | 1 | 1 | 1 | 1 | 1 | 1 | 1 |
| 12. Was appropriate statistical analysis used? | 1 | 1 | 1 | 1 | 0 | 1 | 1 | 1 |
| 13. Was the trial design appropriate, and any deviations from the standard RCT design (individual randomization, parallel groups) accounted for in the conduct and analysis of the trial? | 1 | 1 | 1 | 1 | 1 | 1 | 1 | 1 |
| **JBI Quality score (REF)** | 11/13 | 10/13 | 11/13 | 8/13 | 10/13 | 10/13 | 9/13 | 10/13 |
|  | 85% | 77% | 85% | 62% | 77% | 77% | 69% | 77% |
| **Level of evidence (REF)** | 1.c | 1.c | 1.c | 1.c | 1.c | 1.c | 1.c | 1.c |
